# Referee Acknowledgement for 2010

**DOI:** 10.1038/bjc.2011.195

**Published:** 2011-06-07

In this issue, we publish the names of those who reviewed manuscripts for us in 2010.

The Editor-in-Chief, Specialist Editors and everyone involved in publishing *BJC* would like to extend our sincere thanks to them for contributing their expertise and time. Our referees play an invaluable role in ensuring that *BJC* continues to publish the high-quality original papers and reviews that make it one of the world's leading oncology journals.

Aaltonen, Lauri A

Aaronson, Neil

Aaronson, Stuart

Abbott, BL

Abdel-Wahab, M

Abe, Takashige

Acero, Nuria

Achen, Marc G

Adachi, Yasushi

Adamo, Sergio

Adams, Richard

Adriana, Albini

Agalliu, Ilir

Aggarwal, Amita

Ahern, Roger

Ajani, Jaffer

Alberts, Steven R

Alfirevic, Ana

Allen, Naomi E

Allgood, Prue C

Allory, Yves

Alonso, Javier

Altevogt, P

Altundang, Karim

Amann, Anton

Amato, Robert

Amir, Eitan

Amos, Christopher I

Anders, Carey K

Anderson, Peter

Andersson, Mattias

Andre, Fabrice

Andre, Thierry

Aneja, Ritu

Annabi, Borhane

Annunziata, CM

Anthoney, Alan

Antonarakis, Emmanuel S

Aoki, Taku

Aprile, Giuseppe

Arafat, Hwyda A

Arbuthnot, Patrick

Arciniegas, E

Arias-Pulido, Hugo

Arkenau, Hendrik-Tobias

Armes, Jane

Armes, Jo

Armstrong, Michael

Armstrong, Miranda

Arnold, R

Artz, Andrew S

Arus, Carles

Ashing-Giwa, Kimlin T

Ashworth, Alan

Asmis, Timothy

Attard, Gerhardt

Atzori, Francesco

Aubele, Michaela

Augustine, Christi K

Auranen, Annika

Avila, Matias A

Awsare, Ninaad

Babal, Pavel

Babiera, Gildy

Bachmann, M

Badve, Sunil

Bae, Insoo

Baffa, Raffaele

Bagnoli, Marina

Baier, PK

Baker, AF

Baker, Frank

Balkwill, Fran

Bamias, Aristotle

Baracos, Vickie E

Baral, Rathindranath

Baranowska-Kortylewicz, Janina

Barberi-Heyob, Muriel

Barbolina, Maria

Bardelli, Alberto

Bar-Eli, Menashe

Baron, Daniel

Bar-Sela, Gil

Bartek, J

Basu, Sandip

Bates, David O

Batra, Raj K

Batra, Surinder K

Bauer, Stefan

Bay, BH

Baynes, RD

Beaver, Kinta

Beer, Ambros J

Begent, RHJ

Beijnen, Jos H

Beliën, JAM

Belinsky, Steven A

Belldegrun, A

Bellone, Stefania

Bendayan, Reina

Benedix, Frank

Benlloch, Susana

Bennett, Charles

Bennett, Rachel

Berchem, Guy

Berchtold, Martin W

Bergenmar, Maria

Bergmann, Lothar

Bernier, Gilbert

Bernstein, J

Berstein, Lev

Bertheau, Philippe

Bertolini, Federica

Bertolini, Francesco

Berwick, Marianne

Besse, Benjamin

Besser, Michal J

Bhagwat, Ashok S

Bhatia, M

Bhoola, KD

Biagi, Ettore

Bianco, Roberto

Bigner, DD

Bing, Chen

Bingle, Lynne

Birkenkamp-Demtroder, Karin

Bishop-Bailey, D

Bisogno, Gianni

Björkman, M

Black, EP

Blamey, Roger

Blanchetot, C

Blanco, Javier C

Blay, Jean-Yves

Blettner, Maria

Bloomston, Mark

Boccardo, E

Bocchetta, Maurizio

Bocci, Guido

Bode, B

Bodmer, Walter

Boerner, Julie

Boffetta, Paolo

Boise, Larry

Boland, C Richard

Bonnet, Dominique

Bonvalot, Sylvie

Boormans, Joost

Borst, Piet

Bosaeus, Ingvar

Bosari, Silvano

Bosch, Francesc

Bosetti, Cristina

Bosman, FT

Bouchahda, Mohamed

Boucheix, Claude

Bourdel-Marchasson, Isabelle

Bours, Vincent

Boven, Epie

Bower, Mark

Boyd, Norman F

Braakhuis, Boudewijn

Brackmann, Stephan

Brada, Michael

Bradbury, Penelope A

Brase, JC

Brauch, Hiltrud

Brekken, Rolf

Brennan, Bernadette

Brenner, Hermann

Bridgewater, John

Bronner, Christian

Brown, Janet E

Brown, Robert

Brufsky, Adam

Brumatti, Gabriela

Bruno, Savino

Bucci, Barbara

Buccione, Roberto

Buchler, Markus W

Buchsbaum, Donald

Buckland, G

Budd, G Thomas

Budhram-Mahadeo, Vishwanie

Budillon, Alfredo

Bukowski, Ronald

Buonaguro, Franco M

Burdon, Tom G

Burgdorf, Stefan

Burger, Angelika

Burris, Howard

Bushell, Martin

Cabibbo, G

Cai, Tommaso

Cairns, Ben

Calandrino, Riccardo

Calin, George

Cameron, David

Campbell, Helen E

Campbell, Moray J

Camussi, Giovanni

Caraglia, Michele

Carbone, Michele

Cardoso, Fatima

Carducci, Michael

Carlsson, Sigrid

Carmeliet, Peter

Carpenter, Lucy

Carpino, Nicholas

Carr, DW

Carraro, Dirce

Cascinu, Stefano

Cassidy, Aedin

Cassidy, Jim

Castresana, Javier

Catalano, Vincenzo

Catchpoole, Daniel R

Ceppi, Paolo

Chakrabarty, Subhas

Chakravarti, Arnab

Chalmers, A

Chamie, Karim

Chamorey, Emmanuel

Chan, Andrew T

Chan, David W

Chan, Stephen

Chang, Alex

Chang, Wen-Chi

Chao, Tsu-Yi

Chatelut, Etienne

Chau, Ian

Chauhan, Subhash C

Chen, Allen M

Chen, Chang-Yan

Chen, J

Chen, Johnny

Chen, Kong Y

Chen, Miao-Fen

Chen, Shaoyong

Chen, Shi-Shi

Chen, Shu-Jen

Chen, Thomas C

Chen, Wei

Chen, Xueyu

Chenevix-Trench, Georgia

Cheng, Ann-Joy

Cheng, Iona

Cheng, Ji-Ming

Cheng, Jonathan

Chew, Helen

Chiarion-Sileni, Vanna

Chieffi, Paolo

Cho, Nam Hoon

Choe, Wonchae

Choi, H

Chow, Nan-Haw

Christmas, SE

Chua, Daniel

Chuthapisith, S

Ciruelos, EM

Ciudad, Carlos

Clamp, Andrew R

Clark, Peter

Clarke, Robert

Clarke, Stephen J

Clarkin, Claire

Clavel, Christine

Clevenger, Charles

Cochran, Alistair

Cohen, Ilan

Cohen, Stanley N

Coleman, Robert

Coley, Helen M

Colotta, Francesco

Comoli, P

Constantinidou, Anastasia

Cooling, Laura

Cooper, Colin S

Coppola, Domenico

Cornud, F

Correa, Pelayo

Corrie, Pippa G

Corsello, SM

Corso, Alessandro

Cory, Giles

Costelli, Paola

Coutant, Charles

Coutlee, Francois

Cox, Brian

Coyne, James

Cross, Amanda J

Cruickshank, Margaret E

Crystal, RG

Culig, Zoran

Cunningham, David

Curran, Des

Cuzick, Jack

Dahl, Olav

Dahlberg, Suzanne

Dahut, William L

Dal Maso, Luigino

Dalay, Nejat

Dalgleish, Angus G

Damato, Bertil E

Dame, Christof

Dancey, Janet

Dangoor, Adam

Danson, S

Darby, Sarah

Darley, Richard

Das, Bikul

Das, Kumuda

Davey, Ross

Davies, Faith

Davies, Michael A

Dayao, Zoneddy

De Boer, Angela

De Bono, Johann S

De Clercq, Wim

De Gruijl, Tanja D

De Ponti, Fabrizio

De Vries, EGE

Debiec-Rychter, Maria

DeClerck, Yves A

Degani, Hadassa

Deissler, Helmut

Delahanty, Douglas L

Delorme, Stefan

Deltour, I

Denhardt, David T

Denkert, Carsten

Dent, Paul

Deonarain, Mahendra P

Desbois-Mouthon, Christèle

Desmedt, Christine

Detterbeck, Frank

Detti, Beatrice

Di Renzo, Maria Flavia

Dias, Sérgio

Diasio, Robert

Diaz, Bruno L

Diener-West, M

Dimopoulos, MA

D’Incalci, Maurizio

Ding, Yan

Dingemans, Anne-Marie C

Dive, Caroline

Dixon, J

Dizdar, Omer

Dobrzanski, Mark J

Dobson, Hilary

Dodwell, David

Doherty, Glen

Doi, Toshihiko

Doll, Andreas

Donato, Rosario

Dong, Jin-Tang

Donnem, T

Dotti, Gianpietro

Dou, Q Ping

Dowlati, Afshin

Driessen, C

Dudley, Mark

Duffy, Stephen W

Dunlop, David

Dunlop, Malcolm

Durham, William

Duric, Nebojsa

Dvorchik, Igor

Dyer, Martin

Eastman, Alan

Edwards, Paul

Efficace, Fabio

Eggly, Susan

Einarsdóttir, Kristjana

Eisen, Tim

Elder, David E

Elisei, R

Elit, Laurie

El-Kareh, AW

Ellenson, LH

Ellinger, Jörg

Ellis, Ian

Ellis, Lee M

Elmore, Joann

El-Naggar, Adel

Emdin, Michele

Emmenegger, Urban

Enver, Tariq

Epstein, JB

Epstein, Richard

Ernst, Edzard

Eskens, Ferry ALM

Esposito, Franca

Evans, D Gareth R

Ewertz, Marianne

Fabregat, Isabel

Fakih, Marwan

Falandry, Claire

Falanga, Anna

Falasca, Marco

Falk, Stephen

Fan, J

Fantini, Massimo C

Farrar, CT

Fearon, Eric

Fearon, Kenneth CH

Fedier, Andre

Fedorocko, Peter

Fehm, Tanja

Feig, Barry

Feliu, Jaime

Fenner, Martin H

Fenton, Jenifer I

Feo, Francesco

Feron, Olivier

Feun, L

Fiander, Alison

Field, John K

Figlin, Robert

Filmus, Jorge

Finn, Richard S

Fisher, PB

Flaherty, Keith

Fleisher, Martin

Fleming, Gini

Fleming, Jean S

Fletcher, Olivia

Fock, Kwong Ming

Foekens, John A

Font, Albert

Forbes, Lindsay

Foster, Christopher

Foukakis, Theodoros

Foulkes, William

Franc, Brigitte

Franceschi, Silvia

Franchi, Alessandro

Francken, Anne B

Franco, Eduardo

Frattini, Milo

Fregnani, José

French, Samuel W

Friess, Helmut

Froehner, Michael

Frost, Andra R

Fugazzola, Laura

Fujii, Yasuhisa

Fukuda, Seiji

Fukushima, Tsuyoshi

Fulda, Simone

Funamizu, Naotake

Furuse, Junji

Fusco, Alfredo

Futreal, P Andy

Gagnon, Bruno

Galaal, Khadra

Galanaud, JP

Galanti, Rosaria

Galietta, Luis JV

Gallagher, Michael

Gallick, Gary E

Gallouzi, IE

Galmarini, Carlos Maria

Galustian, C

Gann, PH

Ganz, Patricia

Gao, G

Gao, Jian-Xin

Garcia Martin, Margarita

Garcia, Jorge A

Garcia-Lora, Angel M

Garraway, Levi A

Garrido, Federico

Garrod, David

Gatta, Gemma

Gatter, Kevin C

Gautam, Subhash C

Gazzaniga, Paola

Gebski, Dr VJ

Gelderblom, Hans

Geller, Alan

Geller, David A

Gemmill, Robert

Gennari, Alessandra

Geoerger, B

Gespach, Christian

Ghaneh, Paula

Ghatak, Shibnath

Ghosh, Sourav

Giaccone, Giuseppe

Giannelli, Gianluigi

Gianni, Luca

Giantonio, Bruce

Gil, Ziv

Giles, Francis

Gilham, David

Gilliam, AD

Gilloteaux, J

Ginsberg, Jill

Gioeli, D

Giralt, Jordi

Gires, Olivier

Given-Wilson, Rosalind

Gnant, Michael

Goldenberg, David

Goldstein, David

Goldwasser, François

Gollin, Susanne M

Gorgoulis, VG

Goteri, G

Gottesman, Michael

Gottschalk, Stephen

Gown, Allen M

Graff, Jeremy

Greaves, Mel

Green, Jane

Green, John A

Greggi, Stefano

Grisanti, Salvatore

Grisel, Jedidiah J

Gronberg, Henrik

Grosche, Bernd

Gross, Mitchell

Gu, Fangyi

Guarneri, Valentina

Guchelaar, Henk-Jan

Guerrero, D

Guerriero, S

Guittet, Lydia

Guix, Marta

Gullick, William J

Guo, ZS

Gupta, Anirban Sen

Gururangan, Sridharan

Gustafson, WC

Gutierrez, Martin E

Gwyther, Steve

Hahn, CA

Haka, AS

Hall, Janet

Hall, Michael

Hamashima, Chisato

Hamblin, Michael R

Hamilton, William

Han, Ji-Youn

Hannaford, Philip

Harmsen, Stefan

Harper, Diane M

Harper, Sam

Harrington, Kevin J

Harry, Vanessa

Harvey, Amanda

Harvey, Kieran

Hassan, Md

Hatake, Kiyohiko

Haun, Randy S

Havrilesky, Laura

Hawk, Ernest T

Hawkins, William G

Hearn, Julie

Hegel, Mark T

Heidenreich, Axel

Heimann, Ruth

Helmig, Simone

Hemler, ME

Hemminki, Kari

Henoch, I

Henry, Norah Lynn

Henschke, Claudia

Hepworth, SJ

Herchenhorn, Daniel

Hermsen, BBJ

Herrero-Martín, David

Hershman, Jerome M

Hes, Frederik

Hess, Kenneth R

Hildebrandt, Gerhard

Hirsh, Vera

Hiscox, Steven

Hochhauser, Daniel

Hoeben, RC

Hoffman, Philip C

Hofheinz, Ralf-Dieter

Hofmann, TG

Hofvind, Solveig

Hogberg, Thomas

Hohenegger, Martin

Holdenrieder, S

Holen, Ingunn

Holmen, Sheri L

Holyoake, Tessa L

Hopper, John L

Horst, David

Houssami, Nehmat

Howell, Stephen B

Høybye, Mette Terp

Hrelia, Patrizia

Hu, Hongbo

Huang, Cheng-Long

Huang, Haojie

Huang, P

Huang, Qin

Huang, Shuang

Huang, Xin

Huddart, Robert A

Huebner, Kay

Huerta, Sergio

Hung, Jaclyn Y

Hutton, Jennifer

Huynh, Hung

Ichikawa, Daisuke

Im, Young-Hyuck

Imyanitov, Evgeny

Inoue, Akira

Inoue, Manami

Irby, Rosalyn

Ishii, Genichiro

Ishii, Hiroshi

Ishioka, Chikashi

Ishizuka, Mitsuru

Ito, Yasuhiro

Ivanov, Vladimir N

Iversen, Lene H

Iwasaki, Motoki

Izzo, Francesco

Jacot, William

Jaeger, Ruy G

James, Aimee S

Jankowski, Janusz

Janneh, O

Jasani, Bharat

Jayson, Gordon C

Jenkins, John T

Jiang, Hao

Jiang, Shi-Wen

Jin, Yening

Jing, Naijie

Joab, Irene

Jodrell, Duncan

Joffe, Steven

Johansson, Birgitta

Johnson, Phillip J

Jones, Chris

Jones, Donald JL

Jones, Frank E

Jones, George DD

Jones, Philip

Jones, Richard B

Jones, Robin

Jonkers, Jos

Joosten, J

Jordanova, ES

Jørgensen, Karsten

Jotte, Robert

Jubb, Adrian M

Juergens, Rosalyn

Jung, Klaus

Jung, Monica

Kabakov, Alexander E

Kakimi, Kazuhiro

Kalemkeria, Gregory P

Kamangar, Farin

Kamen, Barton A

Kanaoka, S

Kaneko, Michio

Kang, Peter M

Kang, Yoon-Koo

Karanikas, V

Karapetis, Christos

Kasamatsu, Takahiro

Kato, Junji

Katoh, Masaru

Kawada, Kenji

Kawamori, Toshihiko

Kawauchi, S

Keeney, Mike

Keep, RF

Kelly, Catherine M

Kelly, Catherine Margaret

Kelly, Karen

Kelly, W Kevin

Key, Timothy

Keyse, Stephen M

Khan, Javed

Khanna, Chand

Khorana, Alok

Kiaris, Hippokratis

Kikuchi, Eiji

Kim, Hee Cheol

Kim, Hong Jin

Kim, Richard

Kim, Tae-You

Kim, Woo Ho

Kim, Young

Kishimoto, Takashi

Kitchener, Henry C

Kiura, Katsuyuki

Kivela, Tero

Kiyono, T

Kluetz, Paul G

Knijnenburg, Theo A

Kobayashi, Hirotoshi

Kobayashi, Yukio

Koch, Uwe

Kohn, Elise C

Kohne, Claus-Henning

Koike, Yuhki

Kokkola, A

Kollmar, Otto

Kondoh, Masuo

Kono, Suminori

Koukourakis, Michael I

Koutras, Angelos K

Koutsilieris, Michael

Krüger, William’

Krainer, Michael

Kratzke, Robert

Krett, Nancy L

Kruh, Gary

Krull, Kevin R

Kufe, Donald

Kuipers, Ernst

Kulka, Janina

Kumar, Shaji

Kume, Tsutomu

Kummar, Shivaani

Kundi, Michael

Kundu, Gopal C

Kuniyasu, Hiroki

Kunnimalaiyaan, Muthusamy

Kuroda, Junya

Kurtz, Jean-Emmanuel

Kwong, YL

Kyzas, Panayiotis

Lackner, Mark R

Lacroix, Marc

Lagios, M

Lagos, Dimitrios

Lai, Chyong-Huey

Lai, Paul

Lakhani, Sunil

Landi, Maria

Lane, Brian R

Langin, Dominique

Langley, Ruth

Larkin, James

Larson, Andrew C

Larsson, Susanna C

Lash, Timothy

Lassen, Pernille

Laurent-Puig, Pierre

Laurier, Dominique

Lavelle, Katrina

Law, Malcolm

Layer, Paul G

Lazar, Alexander

Lazo, Pedro A

Le Tourneau, Christophe

Lebbe, Celeste

Lee, Jae-Kwan

Lee, Jean W

Lee, Jong-Min

Lee, Jun Ho

Lee, Keun-Wook

Lee, Peng

Lee, Yong J

Leinonen, Maarit

Leiper, Alison

Leonard, Pauline

Levi, Francis

Levitskaya, Jelena

Levitzki, A

Lewis, James H

Lewis, Nancy L

Li, Daqing

Li, Wenbin

Liao, S

Liauw, Stanley L

Lieberman, Morton

Liekens, Sandra

Lim, Dae-Sik

Lin, Elaine Y

Lin, Jin-Ching

Lindblom, Annika

Lindemann, Kristina K

Linderholm, Barbro

Lisanti, Michael

Lital, Keinan-Boker

Liu, Fei-Fei

Liu, Ming

Liu, RS

Liu, Shujun

Liu, Tiancheng

Lo Nigro, C

Loblaw, Andrew

Lodder, Wouter L

Logan, Richard FA

Lokeshwar, Vinata

Lončarek, Jadranka

Lorch, Jochen

Lord, SJ

Loric, Sylvain

Löwik, Clemens WGM

Lubbe, Steven

Lubner, SJ

Lucenteforte, Ersilia

Lugli, Alessandro

Lundh, Carina

Luoh, Shiuh-Wen

Lyness, Sarah

Mabjeesh, Nicola J

MacAulay, Calum

MacFarlane, Marion

MacKie, Rona M

Macleod, Una

Madhok, Brijesh M

Madsen, Steen

Maehara, Yoshihiko

Maeurer, Markus

Maggiolini, Marcello

Magliano, Dianna

Magnelli, Lucia

Maher, Eamonn R

Majumdar, AP

Maki, Robert

Maluccio, Mary A

Mancini, Manuela

Mandala, Mario

Manfredi, Sylvain

Maniadakis, Nikos

Manjer, Jonas

Manor, Danny

Manson, M

Marais, Richard

Marcus, Pamela M

Mareel, M

Mariadason, John

Markman, Maurie

Marks, LS

Marosi, Christine

Marsh, Deborah J

Marshall, Ernest

Marsit, Carmen J

Martin, Miguel

Martin-Fontecha, Alfonso

Marzioni, D

Mason, Anne

Master, Viraj

Mastro, Andrea M

Matias-Guiu, Xavier

Matzno, Sumio

Maughan, Tim

Maurel, Joan

Mawrin, Christian

Mayer, Astrid

Mazzanti, Roberto

Mazzolini, Guillermo

McBride, Mary L

McInnes, Keith

McIntosh, Cameron

McKeage, Mark

McKean-Cowdin, R

McLachlan, Sue-Anne

McLendon, Roger E

McLeod, Howard

McMillan, Donald C

Mehnert, Anja

Meier, Pascal

Meijer, Chris JLM

Melero, Ignacio

Melino, Gerry

Mellado, Begoña

Menczer, Joseph

Meng, Songdong

Mentlein, R

Meregalli, C

Merle, Philippe

Meyer, Tim

Meyerhardt, Jeffrey A

Michael, Michael

Michaud, DS

Michieli, Paolo

Michl, Patrick

Middleton, Mark R

Miekisch, Wolfram

Mierke, Claudia

Migliore, Marcello

Milella, Michele

Millikan, Robert C

Milne, Donna

Mindikoglu, Ayse L

Mischel, Paul S

Mitsiades, Constantine

Mitsiades, Nicholas

Mittal, Balraj

Miyajima, Akira

Miyako, Kenichi

Mogensen, Mette M

Mohammad, Ramzi M

Molassiotis, Alexander

Molinari, Michele

Moller, Henrik

Mologni, Luca

Mondal, Debasis

Montazeri, Ali

Montgomery, David Andrew

Moons, KGM

Moore, Malcolm J

Moran, Michael F

Morel, Fabrice

Mori, Yuriko

Morii, Eiichi

Morin, Pat

Morizane, Chigusa

Morrione, Andrea

Morris, Jo

Morris, John C

Moser, Kath

Mosnier, Jean-Francois

Mostert, Bianca

Moulder, Stacy

Mowla, SJ

Moyret-Lalle, Caroline

Muchardt, C

Muenstedt, Karsten

Muggia, Franco

Muñoz-Gámez, José Antonio

Mutch, David

Muwonge, Richard

Mylonas, Ioannis

Nagtegaal, Iris

Naito, Yuji

Nakagawa, Suely Akiko

Nakamura, Yusuke

Nam, Robert

Narod, Steven A

Nasi, Sergio

Neal, David E

Negrini, Massimo

Neoptolemos, John

Neragi-Miandoab, S

Ness, KK

Neugut, Alfred

Neuzil, Jiri

Nevanlinna, Heli

Neven, Patrick

Newman, William G

Newton, Robert

Nghiemphu, Phioanh (Leia)

Nibu, Ken-Ichi

Nicholson, Sandra E

Nieto, MA

Nijman, Hans W

Nijsten, Maarten WN

Nikitin, Alexander Yu

Nilsson, Bengt

Nitti, Donato

Nonn, L

Nordlinger, Bernard

Notario, Vicente

Nothlings, Ute

Nozoe, Tadahiro

Nunes, Everson Araújo

Nutting, Chris M

O’Brien, Mary ER

Ochiai, Atsushi

Ochiya, Takahiro

Odenthal, Margarete

Oeffinger, Kevin

Ogino, Shuji

Oh, Sang-Muk

Ohlmann, Carsten-Henning

Öhrling, Katarina

Ohtsu, Atsushi

Oksenhendler, Eric

Okusaka, Takuji

Oliveria, Susan

Olivotto, Ivo

Olmos, David

Olsen, Anne Helene

Olsen, Catherine

Ooki, Akira

Osada, S

O’Sullivan, Maureen

Ott, Katja

Ottensmeier, Christian

Otto, Thomas

Padhani, Anwar

Paige, Adam JW

Palacios, Jose

Palmqvist, Richard

Pantaleo, Maria Abbondanza

Pantel, Klaus

Paoletti, Xavier

Paolicchi, Aldo

Papadimitriou, Christos A

Papadopoulou, Maria

Paraskeva, Chris

Parikh, Alexander

Park, Ben Ho

Park, Morag

Park, Sang-Yoon

Park, Sook Ryun

Parker, Chris

Parkin, Donald Maxwell

Parkinson, Ken

Paschal, Bryce M

Pasche, Boris

Pastorek, Jaromír

Paszat, Lawrence

Patankar, Manish

Paul, J

Pavlakis, K

Pavlovic, S

Payne, Shannon R

Pazzaglia, Simonetta

Peake, Mick

Pedersen, Christina

Pedley, R Barbara

Peet, Daniel J

Peled, Nir

Pepper, Chris

Perez, Kimberley

Perez-Tomas, Ricardo

Pergolizzi, Joseph

Perkins, Elizabeth

Perona, Rosario

Persson, Jenny

Peters, Godefridus

Peters, Ulrike

Peters, W

Peto, Julian

Petrioli, Roberto

Petry, Karl-Ulrich

Pfannschmidt, Joachim

Pflug, Beth

Pharoah, Paul

Philip, Philip

Phillips, David

Philp, Nancy J

Picard, A Simon

Picelli, Simone

Pichard, Claude

Piché, Alain

Pierga, Jean-Yves

Pignot, Geraldine

Pike, Malcolm C

Pinsky, Paul

Pirie, Kirstin

Pisani, Paola

Pistoia, Vito

Pittet, Valerie

Pittman, Alan

Plentz, Ruben R

Plowman, PN

Plukker, John ThM

Plummer, Elizabeth

Pogribny, Igor P

Pokrovskaja Tamm, Katja

Pölcher, Martin

Polyzos, Aristides

Pompella, A

Poon, Donald

Pornet, Carole

Porta, Camillo

Postma, Aleida

Prabhakar, Uma

Pradhan, Sriharsa

Prestwich, Glenn D

Primrose, John N

Prior, John O

Pritchard, Catrin

Psyrri, Diamando

Qian, Chao-Nan

Quaglia, Alberto

Quinn, David

Quinn, Gwen

Quinten, Chantal

Raanani, Pia

Rabbitts, Pamela H

Rabin, Carolyn

Raez, Louis

Raica, Marius

Ralhan, Ranju

Ramakrishnan, S

Ramalingam, Suresh S

Ramanathan, Ramesh K

Ramirez, Amanda

Ramírez, M

Ramon y Cajal, Santiago

Ramoni, Carlo

Ramos, Maria

Ranstam, Jonas

Rao, Sheela

Rashid, Asif

Ratcliffe, Peter

Rathmell, W Kimryn

Ravaud, Alain

Ray-Coquard, Isabelle

Rayson, Daniel

Reddanna, P

Reddy, Rishindra

Reddy, Sakamuri V

Rédini, Françoise

Redmond, H Paul

Rees, Myrddin

Rego, Eduardo M

Reichrath, Jorg

Reulen, Raoul C

Reynolds, John V

Rhim, Andrew D

Ribatti, Domenico

Ricci, J-E

Richard, Darren E

Richards, Mike

Rihani, Ali

Rimassa, Lorenza

Ringash, Jolie

Rini, Brian

Rishi, AK

Ristimaki, Ari

Robson, Craig

Rocco, James W

Roddam, Andrew W

Roman, Jesse

Romano, MF

Rose-John, Stefan

Rosenberg, Daniel W

Rosenblatt, Karin A

Roskams, T

Roskoski, Robert

Ross, RA

Rosser, Charles

Rossi, Giulio

Rossig, Claudia

Rota, R

Rothe, Francoise

Rottenberg, Sven

Rouviere, Oliver

Rowe, M

Rubinstein, Eric

Rueda, Bo

Ruffini, Enrico

Ruiz, Agustin

Rüschoff, J

Russo, Antonio

Russo, Gian Luigi

Rustin, Gordon JS

Rutella, Sergio

Ruthardt, Martin

Ryder, Steve

Saad, Everardo D

Sach, Tracey H

Sadahiro, Sotaro

Sagae, Satoru

Saito, Hideyuki

Saito, Kazutaka

Sakamoto, Kathleen M

Salama, SA

Saldanha, G

Samali, Afshin

Sampson, John

Samson, Michel

Sanchez-Lara, Karla

Sanderson, Ralph D

Sangro, Bruno

Sankaranarayanan, R

Sanson, Marc

Sant, Milena

Santin, Alessandro D

Santini, Daniele

Sargent, Alex

Sargent, Daniel

Sarkaria, Jann N

Sato, T

Savoldo, Barbara

Sawada, Tokihiko

Scarpa, Aldo

Scartozzi, Mario

Schalken, Jack

Schmetzer, Helga

Schmid, Peter

Schmidtmann, I

Schmitt, Fernando

Schobert, Rainer

Schoffski, Patrick

Schonberg, Mara

Schover, Leslie R

Schulenburg, Axel

Schuuring, Ed

Schuz, Joachim

Schwartz Jr, Simo

Schwartz, EL

Schwartz, Ken

Schwartzbaum, JA

Schwarz, Stephan

Scorilas, Andreas

Segnan, Nereo

Seki, Naohiko

Seliger, Barbara

Serini, Simona

Serrano, Manuel

Seshadri, Mukund

Seshagiri, Somasekar

Sessa, Cristiana

Seto, Takashi

Sgroi, Dennis C

Shah, Manisha

Shao, Z-M

Shi, Huanzhong

Shi, Qian

Shih, Ie-Ming

Shim, Hyunsuk

Shimizu, Chikako

Shimizu, Masahito

Shin, Young Kee

Shinn, Eileen H

Shioda, T

Shiotani, A

Shipley, Janet

Short, Susan

Siddiqi, Azfar-e-Alam

Sidransky, David

Sieber, Oliver

Sikkema, Marjolein

Sikora, Karol

Silber, John

Silver, Andrew

Silvestrini, Rosella

Simard, Julia

Simó, Rafael

Simon, Alice

Simoncini, T

Singal, R

Singh, Piksi

Singh, Rajan

Singh, Salil

Sipahi, I

Siu, Lillian L

Siwak, Doris R

Skipworth, Richard

Skubitz, K

Sleijfer, Stefan

Sluiter, Michelle

Small, Matthew

Smalley, Keiran SM

Smith, Harvey

Smith-McCune, K

Snijders, PJF

Snyder, Claire F

Sobrinho-Simoes, Manuel

Soderberg, Ola

Song, Bo

Sonnemann, Juergen

Soper, SA

Sorbye, Halfdan

Sorensen, Poul HB

Souglakos, John

Spencer, Andrew

Spillman, Monique

Srivastava, Meera

Srivastava, Rakesh

Stacpoole, Peter W

Stanbridge, Eric

Stassi, Giorgio

Stathopoulos, George P

Stattin, Par

Steele, Nicola

Steinberg, Pablo

Steliarova-Foucher, E

Stelzner, Sigmar

Stevens, Richard J

Steward, William P

Stewart, David J

Stiller, Charles

Stocken, Deborah

Stoeber, Kai

Stoeckle, E

Stolley, MR

Stricker, Bruno H Ch

Stummer, Walter

Sullivan, Richard

Suman, Vera

Sun, Shi-Yong

Supiot, Stéphane

Sutherland, Robert

Swanson, John

Swanton, Charles

Sweidan, Michelle

Swindell, Rick

Sylvester, Paul W

Szallasi, Zoltan

Szarvas, Tibor

Szekely, Borbala

Szlosarek, Peter

Szoka, FC

Tabernero, Josep

Taghikhani, Mohammad

Tagliabue, Elda

Taheri-Kadkhoda, Zahra

Tajima, Yusuke

Takahashi, Chiaki

Takano, Shingo

Takashima, Atuso

Takenawa, Tadaomi

Talmadge, James E

Tamura, Kenji

Tang, Yao

Tanner, Stephan M

Tannock, Ian F

Tanvetyanon, Tawee

Taphoorn, Martin JB

Taraboletti, Giulia

Tartour, Eric

Taskila, Taina

Taylor, Catherine

Taylor, JM

Taylor-Robinson, Simon

Tebbutt, Niall Christopher

Teglund, Stephan

Teh, Muy-Teck

Tejpar, Sabine

Teodori, Elisabetta

Terhaar, Jochim S

Terracciano, Luigi

Teschendorf, Christian

Teschendorff, Andrew

Thiery, Jean Paul

Thomas, Charles R

Thomas, JE

Thomas, Nancy E

Thompson, John

Thongprasert, S

Thorne, Rick F

Tisdale, Michael J

To, Shing Shun Tony

Toffoli, Giuseppe

Tofilon, Philip

Tolcher, Anthony W

Tollenaar, Rob AEM

Tomida, Akihiro

Tomlinson, Ian PM

Tomlinson, Mathew

Toriola, Adetunji T

Tortora, Giampaolo

Touloumi, G

Travis, Ruth

Treiber, G

Trivers, KF

Trotti, Andy

Tse, Gary

Tsilidis, Konstantinos

Tulchinsky, E

Ueda, Ryuzo

Ueda, Takeshi

Uematsu, Takayoshi

Uhr, Jonathan

Unger, Florian T

Uren, A

Ursin, Giske

Usuda, Jitsuo

Vacca, Angelo

Vaishampayan, Ulka

Valabrega, Giorgio

Valle, Juan W

Valyi-Nagy, Tibor

Van de Nieuwenhof, Hedwig P

Van den Bent, MJ

Van Duijnhoven, Fränzel

Van Gool, Stefaan

Van Kempen, Leon

Van Laere, Steven J

Van Lier, Rene A

Van Meerbeeck, Jan

Van Zandwijk, Nico

Varma, Abhay K

Varticovski, Lyuba

Vasudev, NS

Vauthey, Jean-Nicolas

Velayos, Fernando

Venkitaraman, Ramachandran

Verhoef, C

Verset, Gontran

Viñals, Francesc

Villa, Erica

Vincenzi, Bruno

Vitale, M

Vivanco, Maria

Vlad, Anda M

Vlodavsky, Israel

Von Eggeling, Ferdinand

Von Wagner, Christian

Vonka, Vladmir

Wagman, Lawrence

Wagner, Lars

Wagner, Oswald

Wagner, Simon

Wakeford, Richard

Walker, Rosemary A

Wallis, Matthew

Wallstrom, Peter

Wands, Jack

Wang, Bo

Wang, Ming-Rong

Wang, Xin Shelley

Wang, Yaohe

Wang, Yufei

Ward, Douglas

Wardle, Jane

Wasserfallen, Jean-Blaise

Watanabe, Maria AE

Watson, Dennis

Watson, Joanna

Watters, Dianne

Webb, Penelope

Wei, Alice C

Weitberg, Alan B

Weizer, Alon Z

Welch, Danny R

Welch, Heidi CE

Weller, David P

Werneke, U

Werner, Haim

Wesierska-Gadek, Jozefa

West, Catharine ML

Westra, TA

White, Bruce

Whiteside, Theresa L

Wilcock, Andrew

Willett, Christopher

Williams, Ann Caroline

Williams, Gareth Haydn

Williams, John HH

Wilop, S

Wilson, Mathew W

Wilson, William

Woetmann, A

Wolpin, Brian M

Wolter, Pascal

Wondrak, GT

Wong, Ling S

Wrensch, Margaret

Wright, Jim

Wright, Margaret E

Wu, Kun

Wun, Ted

Xia, Weiming

Xu, Yan

Yaffe, Martin

Yalcin, Suayib

Yamamoto, Masato

Yamanaka, Ryuya

Yang, Jia

Yang, Pan-Chyr

Yao, Jian

Yashiro, Masakazu

Yee, Douglas

Yeger, Herman

Yeudall, W Andrew

Yoshioka, Takashi

Young, Lawrence

Yousef, George

Yun, Jong K

Zabner, J

Zaffaroni, Nadia

Zahl, Per-Henrik

Zanini, Cristina

Zatloukal, Kurt

Zeimet, AG

Zhang, Dongwei

Zhang, Lin

Zidi, Ines

Ziegler, John L

Zimmermann, Camilla

Zimmers, Teresa A

Zingg, U

Zlobec, Inti

Zong, Wei-Xing

Zorzi, Manuel

Zwerschke, Werner

